# High Expression Levels of SIGLEC9 Indicate Poor Outcomes of Glioma and Correlate With Immune Cell Infiltration

**DOI:** 10.3389/fonc.2022.878849

**Published:** 2022-06-09

**Authors:** Heng Xu, Yanyan Feng, Weijia Kong, Hesong Wang, Yuyin Feng, Jianhua Zhen, Lichun Tian, Kai Yuan

**Affiliations:** ^1^ School of Life Sciences, Beijing University of Chinese Medicine, Beijing, China; ^2^ Shenzhen Bao’an Traditional Chinese Medicine Hospital, Guangzhou University of Chinese Medicine, Shenzhen, China; ^3^ Beijing Hospital of Traditional Chinese Medicine Clinical Medical College, Beijing University of Chinese Medicine, Beijing, China; ^4^ Beijing Research Institute of Chinese Medicine, Beijing University of Chinese Medicine, Beijing, China

**Keywords:** glioma, SIGLEC9, diagnostic marker, mechanism, therapeutic target

## Abstract

**Objective:**

This study aimed to investigate the diagnostic value and underlying mechanisms of sialic acid-binding Ig-like lectin 9 (SIGLEC9) in gliomas.

**Patients and Methods:**

The Cancer Genome Atlas (TCGA) and the Chinese Glioma Genome Atlas (CGGA) databases were used to analyze the association of *SIGLEC9* expression levels with tumor stages and survival probability. Immunohistochemical staining of SIGLEC9 and survival analysis were performed in 177 glioma patients. Furthermore, related mechanisms were discovered about SIGLEC9 in glioma tumorigenesis, and we reveal how SIGLEC9 functions in macrophages through single-cell analysis.

**Results:**

TCGA and CGGA databases indicated that patients with high *SIGLEC9* expression manifested a significantly shorter survival probability than those with low *SIGLEC9* expression. *SIGLEC9* was upregulated significantly in malignant pathological types, such as grade III, grade IV, mesenchymal subtype, and isocitrate dehydrogenase wild-type gliomas. The immunohistochemical staining of tissue sections from 177 glioma patients showed that high-SIGLEC9-expression patients manifested a significantly shorter survival probability than low-SIGLEC9-expression patients with age ≧60 years, grade IV, glioblastoma multiforme, alpha thalassemia/intellectual disability syndrome X-linked loss, and without radiotherapy or chemotherapy. Furthermore, the *SIGLEC9* expression level was positively correlated with myeloid-derived suppressor cell infiltration and neutrophil activation. The *SIGLEC9* expression was also positively correlated with major immune checkpoints, such as *LAIR1*, *HAVCR2*, *CD86*, and *LGALS9*. Through single-cell analysis, we found that the *SIGLEC9* gene is related to the ability of macrophages to process antigens and the proliferation of macrophages.

**Conclusion:**

These findings suggested that SIGLEC9 is a diagnostic marker of poor outcomes in glioma and might serve as a potential immunotherapy target for glioma patients in the future.

## Introduction

Gliomas are primary intra-axial brain tumors representing 80% of all malignant brain tumors (1). Patients with glioma often suffer from vomiting, headaches, seizure, and visual loss. The causes of gliomas include hereditary disorders, radiation, and inherited polymorphisms of DNA repair genes (2). Inherited polymorphisms of DNA repair genes might increase the risk of gliomas (3). DNA damage accumulation would occur when DNA repair gene expression is decreased, which might increase the frequencies of mutation. The classification of gliomas depends on tumor grade, cell type, and tumor location (4). Gliomas could be categorized from World Health Organization (WHO) grade I to WHO grade IV. WHO grade I represents the least advanced disease with the best prognosis, while WHO grade IV represents the most advanced disease with the worst prognosis. In addition, the classification of gliomas depends on histological features, including classical subtype (CL), proneural subtype (PN), mesenchymal subtype (ME), *etc.* (5). The treatment of gliomas is a combined method of radiation, surgery, and chemotherapy (6). The prognosis of gliomas varies from different grades and subtypes. Low-grade glioma patients have better 5- and 10-year survival rates. While high-grade glioma patients have poor survival rates, the median overall survival of glioblastoma multiforme is approximately 15 months (7). Patients with isocitrate dehydrogenase (IDH) 1 or 2 mutated gliomas have a higher survival rate than patients with IDH wild-type gliomas (8).

In recent years, immune checkpoint modulators (ICIs) have been found to be important means of treating cancer, and ICIs targeting CTLA-4 or PD-1/PD-L1 have been developed (9, 10). Sialic acid-binding Ig-like lectins (SIGLECs) act as inhibitory receptors on innate and adaptive immune cells to suppress immune responses, and SIGLEC9 on neutrophils and SIGLEC7 on NK cells can potentially suppress antitumor immune responses (11). SIGLEC9 can be targeted to enhance therapeutic antitumor immunity *in vivo* (12). SIGLEC9 is a putative adhesion molecule mediating sialic-acid-dependent binding to cells (11). It contains a cytoplasmic motif referring to the immunoreceptor tyrosine-based inhibitor motif regulating cellular response. The related pathways of SIGLEC9 are class I MHC-mediated antigen processing and presentation, which is an innate immune response. SIGLEC9 is mainly expressed in peripheral blood leukocytes, such as monocytes, neutrophils, and CD56^+^ NK cells. In mice, the functionally equivalent paralog of SIGLEC9 is Siglec-E. In the inflammatory environment, SIGLEC9 in neutrophils and monocytes could induce apoptosis after generating reactive oxygen species. Previous studies have explored the correlation between SIGLEC9 and cancers. Haas *et al.* found that SIGLEC9 modulates memory CD8^+^ T cells to congregate in the tumor microenvironment of melanoma (13). Stanczak *et al.* discovered that SIGLEC9 was upregulated in the tumor-infiltrating T cells in the tumor microenvironment of colorectal cancer, ovarian cancer, and non-small cell lung cancer (14). A high SIGLEC9 expression in T cells is correlated with a decreased survival prognosis of non-small cell lung cancer patients. Beatson *et al.* demonstrated that mucin MUC1 could regulate the tumor immunological microenvironment that follows the engagement of SIGLEC9 (15). Although the functions of SIGLEC9 were explored in some types of cancer, the diagnosis value and underlying mechanism of SIGLEC9 in glioblastoma multiforme (GBM) have not been investigated. Therefore, we will reveal the effect of SIGLEC9 as an immune checkpoint on the immune microenvironment of glioma and provide a theoretical basis for the further development of immunotherapeutic agents for glioma.

In this study, we assessed the correlation between the clinical characteristics and SIGLEC9 expression in glioma patients. The Cancer Genome Atlas (TCGA) and Chinese Glioma Genome Atlas (CGGA) databases were used to compare the expression of *SIGLEC9*. Then, we measured the expression of SIGLEC9 with immunohistochemical staining on 177 patients with gliomas to validate the results. Gene Set Enrichment Analysis (GSEA) was conducted to reveal the biological functions of SIGLEC9 in gliomas. Lastly, functional enrichment analysis was performed to discover the role of SIGLEC9 in gliomas.

## Methods

### Patients and Samples

A total of 177 patients with gliomas undergoing surgery in Sanbo Brain Hospital Capital Medical University were included in this study. The diagnosis of gliomas for every patient was authenticated with laboratory examination, clinical features, and macroscopic and histological examinations. The characteristics of glioma samples, including grade, subtype, and IDH expression, were estimated by two pathologists specializing in brain tumor disorders. The follow-up information of 177 glioma patients was acquired. The endpoint of this study was defined as overall survival (OS), which is the period from the surgery date to death date without a specified cause of death. OS is a useful index to estimate the prognosis of tumor patients. The follow-up period of all patients ended in April 2019. The informed consent of biomedical research about tissue usage has been signed by every patient with glioma, with the project approved by the ethics committee of Sanbo Brain Hospital Capital Medical University (no. SBNK-2018-003-01). We ranked the 177 patients from low to high, and then we identified the first 25 patients as belonging to the low-SIGLEC9-expression group and the last 26 patients as belonging to the high-SIGLEC9-expression group based on the significant difference in survival between the high-SIGLEC9-expression group and the low-SIGLEC9-expression group. The remaining patients were categorized into the medium- SIGLEC9-expression group. The clinical data of 177 patients are shown in **Supplementary Table S1**.

### Immunohistochemical Staining

The protein levels of SIGLEC9 were examined by immunohistochemical staining. Formalin-fixed paraffin-embedded slides of glioma were baked for 4 h. Then, the slides were deparaffinated with dimethylbenzene and dehydrated with gradient ethanol. Furthermore, the slides were subjected to antigen retrieval with boiled citrate buffer, cyclooxygenase block with 3% hydrogen peroxide, and nonspecific antigen block with 10% goat serum. The primary antibody rabbit anti-human SIGLEC9 (Abcam, Cambridge, MA, USA) was diluted to 1:100 and incubated at 4°C overnight. On day 2, the second antibody, horseradish peroxidase-labeled goat anti-rabbit or mouse, was incubated for 1 h. Then, the slide was stained with 3′-diaminobenzidine reagent and counterstained by hematoxylin. Lastly, the total area of positive expression of SIGLEC9 was evaluated with ImageJ software by two researchers in the list of authors. The best cutoff value was determined by the survminer package of R.

### Bioinformatic Analysis of *SIGLEC9* Expression

The normalized values of fragments per kilobase per million mapped reads of gliomas were obtained from TCGA dataset (https://portal.gdc.cancer.gov) before April 2019. The batch effect of low-grade glioma (LGG) and GBM was removed by “sva” package. The normalized datasets of RNA-Seq were conducted as input. TCGA is a useful dataset to catalog genetic mutations responsible for cancer with the method of bioinformatics and genome sequence. The comparison of SIGLEC9 expressions in normal tissue and glioma was conducted in GEPIA website (http://gepia.cancer-pku.cn/). GEPIA is a web server about cancer and normal gene expression. In addition, we downloaded the mRNA-seq data from the CGGA dataset before October 2019. CGGA is a powerful dataset that stores the data of about more than 2,000 samples from Chinese brain tumor patients. The data of CGGA included mRNA sequencing, mRNA microarray, microRNA microarray, whole-exome sequencing, and the matched patient clinical data. The expression of *SIGLEC9* was compared between different grades and subtypes of gliomas. SPSS20.0 was used to evaluate the statistical significance between different groups.

### Gene Set Enrichment Analysis

In this study, we conducted GSEA to investigate the underlying mechanisms of SIGLEC9 in gliomas. GSEA is a method that provides insights into discovering the biological mechanisms of the genes. The correlation between leukocyte infiltration and *SIGLEC9* expression was conducted by single-sample GSEA (ssGSEA). The correlation between immune-related gene sets and *SIGLEC9* was also conducted by ssGSEA. The ssGSEA is the extension of GSEA to measure the separate enrichment scores of each gene set. The ssGSEA could transform to the profile of gene set enrichment, which allows featuring of the cell state of biological process activities and pathways. The correlation between immune-related gene sets and *SIGLEC9* was visualized in the bioinformatics website (https://www.immport.org/).

### Functional Enrichment Analysis

In this study, we calculated the correlation of genes and *SIGLEC9* by Spearman method. We filtered genes with correlation >0.6. Gene Ontology (GO) analysis was used to conduct the functional enrichment analysis. The Database for Annotation, “clusterProfiler”, and “enrichplot” packages were used to analyze and visualize the results. GO analysis is a useful tool to investigate the biological processes, cellular components, and molecular functions. We selected signaling pathways with false discovery rate (FDR) <0.01 and count >10 from the GO and Kyoto Encyclopedia of Genes and Genomes (KEGG) enrichment results.

### Single-Cell Gene Analysis

The single-cell sequencing data of tumor tissues and adjacent tissues of glioma were downloaded from the GSE162631 dataset of the GEO database. We selected 4 tumor samples and 1 paracancerous tissue sample for single-cell transcriptome analysis. Raw gene expression matrices were imported and processed using the Seurat R package. The cells and genes with poor quality were filtered out. The genes expressed in at least 3 cells and high-quality cells with more than 200 genes and less than 5,000 genes were selected for the subsequent analysis. Low-quality cells containing more than 10% mitochondrial genes were excluded. Principal component analysis (PCA) with “FindNeighbors” and “FindClusters” functions was used to perform Uniform Manifold Approximation and Projection (UMAP) to screen the significant top 20 principal components. Then, we clustered the cells with a resolution of dim = 30 and visualized the clustering results using a UMAP scatterplot. Then, the different clusters were annotated to cell types based on the typical marker genes of the cells. Furthermore, we analyzed the expression of SIGLEC9 genes in tumor tissue and adjacent tissue. The ggplot2 package was used to identify the ratio of cells in tumors with adjacent tissue. We re-analyzed the macrophages separately to assess the subtypes of specific cell populations. We used Seurat standard procedures, including PCA dimensionality reduction and tSNE cell construction of clusters to extract cell subsets. Finally, we divided the macrophages into the high-SIGLEC9-expression group and the low-SIGLEC9-expression group according to the median of SIGLEC9 expression. The regulatory role of SIGLEC9 in macrophages was further analyzed.

### Statistical Analysis

Statistical analysis was conducted by *t*-test and Spearman *χ* ^2^ test. Kaplan–Meier analysis was used to evaluate the survival rates. Cox proportional hazard model analysis was conducted to measure the hazard ratio and 95% confidence interval associated with *SIGLEC9* expression. Statistical significance was considered when *P <*0.05.

## Results

### The Expression of *SIGLEC9* in Gliomas With TCGA and CGGA Databases

**Figure 1:**
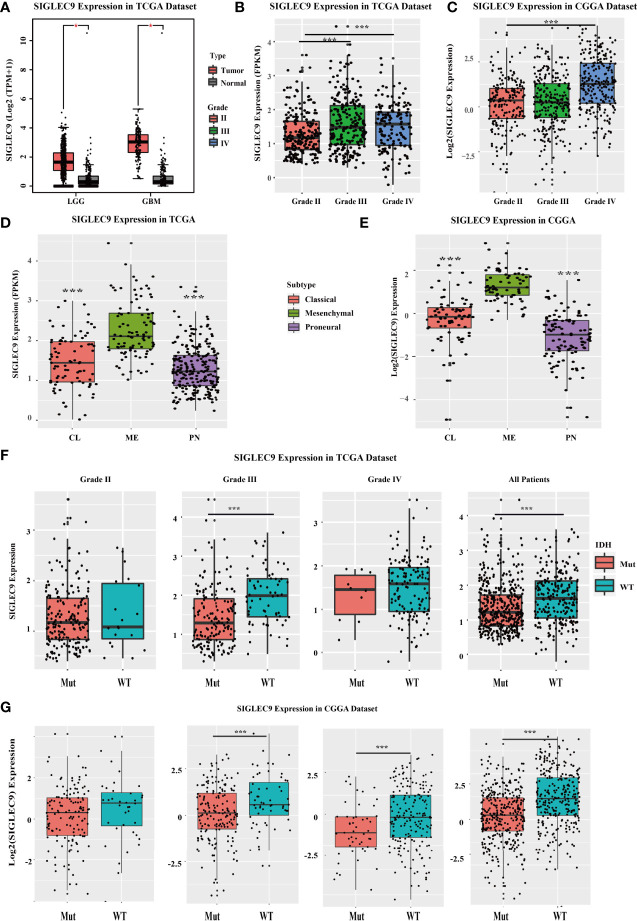
The SIGLEC9 expression in different grades and subtypes with The Cancer Genome Atlas (TCGA) and the Chinese Glioma Genome Atlas **(CGGA)** databases. **(A)** The expression of SIGLEC9 in low-grade glioma (LGG) and glioblastoma multiforme (GBM) with TCGA database. In LGG patients, SIGLEC9 expression was higher in tumor tissue than in adjacent normal tissue (*<0.05). In GBM patients, SIGLEC9 expression was higher in tumor tissue than in adjacent normal tissue (*<0.05). **(B)** The SIGLEC9 expression in grade III patients was higher than in grade II patients with TCGA database (***<0.001). The SIGLEC9 expression in grade IV patients was higher than in grade II patients with TCGA database (***<0.001). **(C)** The SIGLEC9 expression in grade IV patients was higher than in grade II patients with CGGA database (***<0.001). **(D)** The SIGLEC9 expression in the mesenchymal subtype was higher than in the classic and proneural subtypes with TCGA database (***<0.001). **(E)** The SIGLEC9 expression in the mesenchymal subtype was higher than in the classic and proneural subtypes with CGGA database (***<0.001). **(F)** In grade II glioma patients with TCGA database, SIGLEC9 expression was higher in isocitrate dehydrogenase **(IDH)** wild-type patients than in IDH mutated patients (***<0.001). In all glioma patients with TCGA database, SIGLEC9 expression was higher in IDH wild-type patients than in IDH mutated patients (***<0.001). **(G)** In grade II and III glioma patients with CGGA database, SIGLEC9 expression was higher in IDH wild-type patients than in IDH mutated patients (***<0.001). In all glioma patients with CGGA database, SIGLEC9 expression was higher in IDH wild-type patients than in IDH mutated patients (***<0.001).

We investigated the *SIGLEC9* expression in different grades and subtypes of glioma patients with TCGA and CGGA databases. In TCGA database, SIGLEC9 expression was higher in tumor tissue than in adjacent normal tissue in LGG and GBM (**Figure 1A**). The expression of *SIGLEC9* in grade III and grade IV glioma patients was higher than in grade II glioma patients (**Figure 1B**). As for the four subtypes, including classic, mesenchymal, and proneural subtypes, *SIGLEC9* expression was higher in the mesenchymal subtype than in the other two subtypes of gliomas in TCGA database (**Figure 1D**). IDH is regarded as an index for the survival prognosis of gliomas. The results of the TCGA database showed that *SIGLEC9* expression was higher in IDH wild-type glioma patients than in IDH mutated glioma patients (**Figure 1F**). The expression patterns of *SIGLEC9* in different grades and subtypes of glioma patients from the CGGA database were similar to that of TCGA database. In the CGGA database, the expression of *SIGLEC9* in grade IV glioma patients was higher than in grade II patients (**Figure 1C**). The *SIGLEC9* level was higher in the mesenchymal subtype than in the other two subtypes of gliomas (**Figure 1E**). The *SIGLEC9* expression was higher in IDH wild-type gliomas than in IDH mutated gliomas (**Figure 1G**).

**Figure 2:**
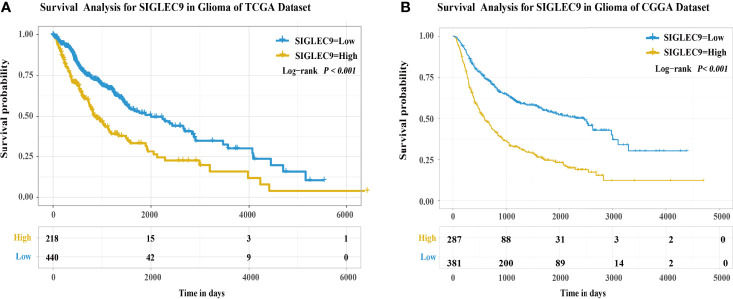
The expression of SIGLEC9 in gliomas with The Cancer Genome Atlas (TCGA) and the Chinese Glioma Genome Atlas **(CGGA)** databases. **(A)** In TCGA database, the glioma patients with a high SIGLEC9 expression (*n* = 218) had a shorter survival probability than those patients with a low SIGLEC9 expression (*n* = 440) (*P* < 0.001). **(B)** In the CGGA database, the glioma patients with a high SIGLEC9 expression (*n* = 287) had a shorter survival probability than those patients with a low SIGLEC9 expression (*n* = 381) (*P* < 0.001).

Furthermore, we used TCGA and CGGA databases to analyze the survival probability of glioma patients with different expression levels of *SIGLEC9*. In TCGA dataset, patients with a higher *SIGLEC9* expression had less survival probability than patients with a lower *SIGLEC9* expression (*P* < 0.001) (**Figure 2A**). In the CGGA dataset, patients with a higher *SIGLEC9* expression also had less survival probability than patients with a lower *SIGLEC9* expression (*P* < 0.001) (**Figure 2B**). Furthermore, Survival analysis in different subgroups with glioma between the high-SIGLEC9 and low-SIGLEC9 groups was performed, the result was shown in **Figure S1**.

### The Expression of SIGLEC9 in Glioma Patients With Immunohistochemical Staining

**Figure 3:**
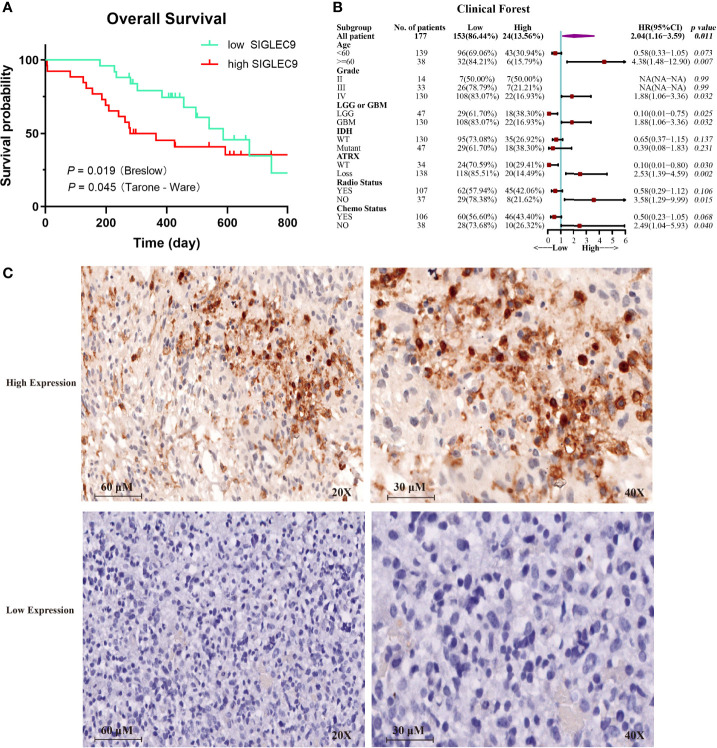
The expression of SIGLEC9 in 177 glioma patients. **(A)** The glioma patients with a high SIGLEC9 expression (*n* = 26) had a shorter survival probability than those patients with a low SIGLEC9 expression (*n* = 25) (*P* = 0.019). **(B)** Clinical characteristics of SIGLEC9 expression in glioma patients. In glioma patients with the following characteristics—age ≧60 years (*P* = 0.007), grade IV (*P* = 0.032), glioblastoma (*P* = 0.032), alpha thalassemia/intellectual disability syndrome X-linked loss (*P* = 0.002), without radiotherapy (*P* = 0.015), or without chemotherapy (*P* = 0.040), respectively—those with a high SIGLEC9 expression had a shorter survival probability than those with a low SIGLEC9 expression. **(C)** Immunohistochemical staining of SIGLEC9 from glioma patients of Sanbo Brain Hospital Capital Medical University.

We analyzed the expression of SIGLEC9 in 177 glioma patients with immunohistochemical staining. Based on the level of SIGLEC9 expression, the glioma patients were divided into three groups, and the clinical data of 177 patients are shown in **Supplementary Table S1**. As shown in **Figure 3A**, patients with a high SIGLEC9 expression manifested a significantly shorter survival probability than those patients with a low SIGLEC9 expression (*P =* 0.019). Then, we analyzed the relationship between clinical characteristics and SIGLEC9 expression in glioma patients. We found that, in glioma patients with age ≧60 years, high-SIGLEC9-expression patients presented a shorter survival probability than low-SIGLEC9-expression patients (*P* = 0.007). Similar phenomena were also observed in patients with grade IV or glioblastoma (*P* = 0.032), patients with ATRX loss glioma (*P* = 0.002), patients without radiotherapy (*P* = 0.015), or patients without chemotherapy (*P* = 0.040). The detailed information on the clinical forest is listed in **Figure 3B**. The immunohistochemical map of glioma patients is shown in **Figure 3C**, and it can be seen intuitively that there are apparent differences in the expression of SIGLEC9 between glioma patients.

### The Correlation Between Immune Microenvironment and *SIGLEC9* Expression in Gliomas

**Figure 4:**
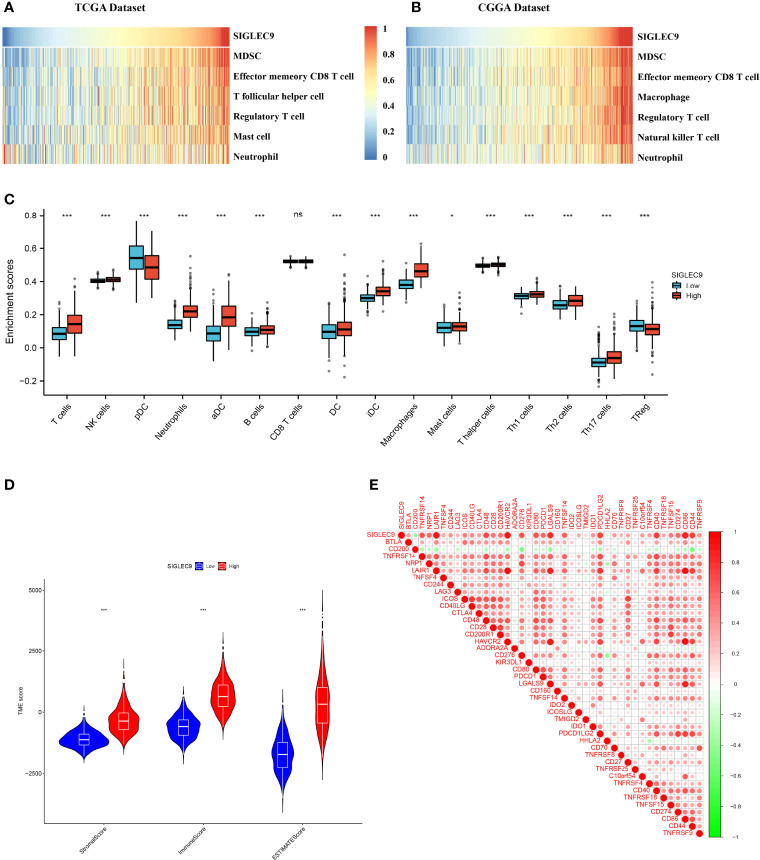
Relationship between SIGLEC9 expression and immune microenvironment. **(A)** In glioma patients with The Cancer Genome Atlas (TCGA) database, SIGLEC9 expression was positively correlated with immune cell infiltration, including myeloid-derived suppressor cells (MDSCs), effector memory CD8 T cells, T follicular helper cells, regulatory T cells, macrophages, *etc.* **(B)** In glioma patients with TCGA database, SIGLEC9 expression was positively correlated with MDSCs, effector memory CD8 T cells, macrophages, regulatory T cells, natural killer T cells, neutrophils, *etc.* **(C)** Differences in immune cell infiltration between the high and the low expression of SIGLEC9, respectively. **(D)** Relationship between SIGLEC9 expression and immune microenvironment score. **(E)** Relationship between SIGLEC9 expression and immune checkpoints. ns, p≥0.05; *, p< 0.05; ***, p<0.001.

In this study, we investigated the correlation between the immune microenvironment and the SIGLEC9 levels in gliomas. Firstly, we analyzed the correlation between immune cell infiltration and *SIGLEC9* expression in gliomas with TCGA database. As shown in **Figure 4A**, the *SIGLEC9* expression was mostly positively correlated with myeloid-derived suppressor cell (MDSC) infiltration (Cor = 0.84, *P* < 2.2e−16), effector memory CD8^+^ T cell infiltration (Cor = 0.71, *P* < 2.2e−16), T follicular helper cell infiltration (Cor = 0.67, *P* < 2.2e−16), regulatory T cell infiltration (Cor = 0.63, *P* < 2.2e−16), mast cell infiltration (Cor = 0.58, *P* < 2.2e−16), and neutrophil infiltration (Cor = 0.27, *P* < 9.6e−13) in gliomas. The CGGA database was also used to explore the correlation between immune status and *SIGLEC9* expression, and similar results were observed. The *SIGLEC9* expression was positively correlated with MDSC infiltration (Cor = 0.88, *P* < 2.2e−16), effector memory CD8^+^ T cell infiltration (Cor = 0.79, *P* < 2.2e−16), macrophage infiltration (Cor = 0.79, *P* < 2.2e−16), regulatory T cell infiltration (Cor = 0.77, *P* < 2.2e−16), natural killer T cell infiltration (Cor = 0.77, *P* < 2.2e−16), and neutrophil infiltration (Cor = 0.55, *P* < 2.2e−16) (**Figure 4B**).

As shown in **Figure 4C**, glioma patients with high SIGLEC9 expression have significantly enhanced infiltration of immune cells, such as T cells, NK cells, neutrophils, aDC, B cells, DC cells, iDC cells, macrophages, mast cells, Th1, Th2, Th17, and so on. The proportion of these immune cells infiltrated was significantly reduced (pDC and Treg). We also calculated the difference in microenvironment scores between the high-SIGLEC9-expression group and the low-SIGLEC9-expression group by the ESTIMATE algorithm, and the results showed that the high-SIGLEC9-expression group had a higher immune score in StromalScore, ImmuneScore, and ESTIMATEScore (*P* < 0.001) (**Figure 4D**).

Then, we evaluated the expression correlation between SIGLEC9 and checkpoint members in tumor-induced immune response using Pearson correlation analysis with TCGA dataset. As shown in **Figure 4E** and **Supplementary Table S3**, SIGLEC9 had a high concordance with LAIR1 (Cor = 0.919, *P* < 0.001), HAVCR2 (Cor = 0.892, *P* < 0.001), CD86 (Cor = 0.870, *P* < 0.001), and LGALS9 (Cor = 0.810, *P* < 0.001).

### Functional Enrichment Analysis of *SIGLEC9*-Correlated Genes

**Figure 5:**
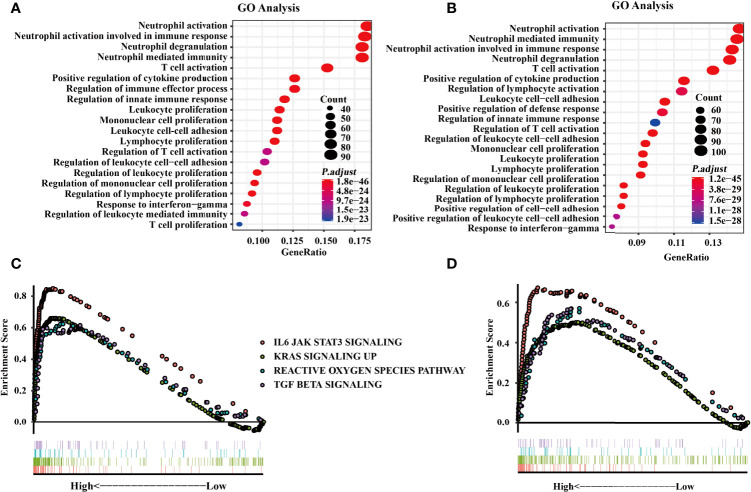
Functional enrichment analysis with The Cancer Genome Atlas (TCGA) and the Chinese Glioma Genome Atlas **(CGGA)** databases. **(A)** Gene Ontology (GO) analysis from the TCGA database. **(B)** GO analysis from CGGA databases. **(C)** Gene Set Enrichment Analysis (GSEA) was conducted to investigate the biological functions of SIGLEC9 in gliomas from the TCGA database. **(D)** GSEA was conducted to investigate the biological functions of SIGLEC9 in gliomas from the CGGA database. The red lines represent SIGNALING as IL6 JAK STAT3. The green line represents KRAS signaling up. The turquoise line represents the reactive oxygen species pathway. The purple line represents TGF-β signaling.

GO analysis was conducted to investigate the functions of *SIGLEC9*-correlated genes in data obtained from TCGA and CGGA databases. Based on the data of TCGA database, GO analysis showed that the *SIGLEC9*-correlated genes were mostly enriched in neutrophil activation, neutrophil degranulation, and neutrophil-mediated immunity (**Figure 5A**). The GO analysis based on the data of CGGA database also showed similar results (**Figure 5B**).

Moreover, GSEA was conducted to explore the biological functions of SIGLEC9 in gliomas. Based on the data of TCGA database, SIGLEC9 was positively correlated with the IL6-JAK-STAT3 signaling pathway (NES = 2.814, FDR = 0), KRAS signaling pathway (NES = 2.474, FDR = 0), reactive oxygen species pathway (NES = 1.896, FDR = 0), and TGF-β signaling pathway (NES = 1.928, FDR = 0) (**Figure 5C**). Similar results were obtained from the GSEA analysis based on the data of the CGGA database (**Figure 5D**), such as IL6-JAK-STAT3 signaling pathway (NES = 2.073, FDR = 0), KRAS signaling pathway (NES = 1.622, FDR = 0.001), reactive oxygen species pathway (NES = 1.668, FDR = 4.40E-04), and TGF-β signaling pathway (NES = 1.700, FDR = 2.91E-04).

### Single-Cell Analysis of Tumor Tissue and Adjacent Tissue

**Figure 6:**
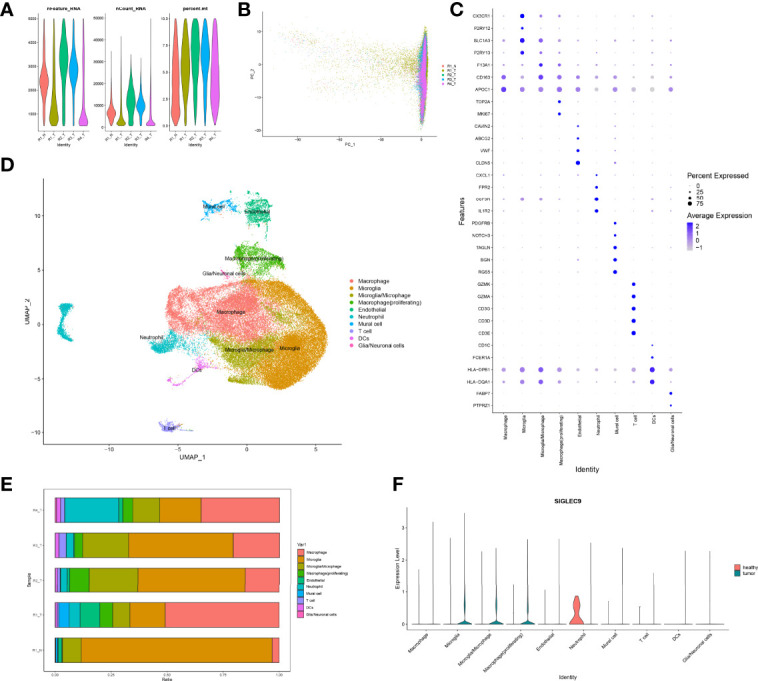
Identification of differentially expressed genes and cell subsets in glioma with single-cell analysis. **(A)** High-quality cell filtration. The cells were filtrated with the number of genes in cells nFeature >200 and ≤5,000; the proportion of mitochondrial genes in cells (percent.mt <10). **(B)** Integration of all samples. **(C)** Bubble plot of marker gene expression in the identification of different cell types. **(D)** Exhibition of cell subsets. **(E)** Proportion of cells in each sample. **(F)** Expression of SIGLEC9 in tumor tissue and in adjacent tissue.

Firstly, we normalized and pooled single-cell data from all samples and filtered the low-quality cells (**Figures 6A, B**). Then, we merged the tumor and adjacent tissue sample to perform unsupervised clustering to identify distinguished cell populations. Seurat v3.0 with default parameters was conducted in this study. We classified different cell subsets according to the related typical marker genes in cells. We mainly identified 10 types of cells (**Figures 6C, D**), such as glial/neuronal cells (PTPRZ1 and FABP7), DCs (HLA-DQA1, HLA-DPB1, FCER1A, and CD1C), T cells (CD3E, CD3D, CD3G, GZMK, and GZMA), mural cell (RGS5, BGN, TAGLN, NOTCH3, and PDGFRB), neutrophil (IL1R2, CSF3R, FPR2, and CXCL1), endothelial (CLDN5, VWF, ABCG2, and CAVIN2), macrophage (proliferating) (MKI67 and TOP2A), macrophage (APOC1), microglia/microphage (CD163 and F13A1), microglia (P2RY13 and SLC1A3), and microglia (P2RY12 and CX3CR1). We calculated the proportion of various types of cells in each sample and found that the proportion of microglia in normal samples was significantly higher than that in tumor samples, but the proportion of macrophages was significantly lower than that in tumor samples (**Figure 6E**). Therefore, our further analysis also focused on macrophages in tumors. Finally, we calculated the expression of SIGLEC9 in these cell subsets between glioma tissue and adjacent tissue (**Figure 6F**). In macrophages (proliferating) and macrophages subsets, the expression of SIGLEC9 in cancer tissues was higher than that in adjacent tissues. Macrophages play an essential role in glioma, and SIGLEC9 may be an important regulator of macrophages.

### Regulatory Role of SIGLEC9 in Macrophages and Macrophages (Proliferating) With Glioma

**Figure 7:**
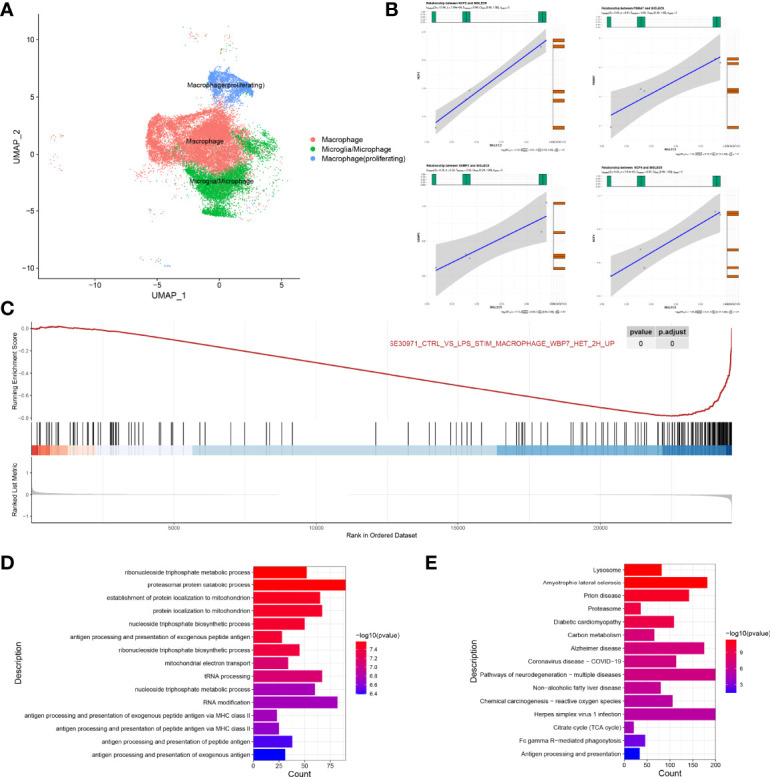
Regulatory role of SIGLEC9 in macrophages with glioma. **(A)** Uniform Manifold Approximation and Projection plot of macrophage cluster. **(B)** Correlation between SIGLEC9 and macrophage activation genes (NCF2, PSMA7, VAMP3, and NCF4). **(C)** Gene Set Enrichment Analysis between high- and low-SIGLEC9 groups in macrophages. **(D)** Gene Ontology analysis between high- and low-SIGLEC9 groups in macrophages. **(E)** Kyoto Encyclopedia of Genes and Genomes analysis between high- and low-SIGLEC9 groups in macrophages.

We extracted a subset of macrophages (macrophage) for analysis (**Figure 7A**), and we found that SIGLEC9 was positively correlated with genes (NCF2, PSMA7, VAMP3, and NCF4) (*P* < 0.05) that are involved in antigen processing and presentation of antigen (**Figure 7B**). Based on the median of SIGLEC9 expression, we divided the macrophages into two groups with high and low SIGLEC9 expression, and we further identified differential genes between these two groups. The GSEA analysis found that differential genes were associated with macrophage activation (16) (**Figure 7C**). The GO enrichment analysis found that upregulated differential genes were significantly enriched in pathways such as antigen processing and presentation of peptide antigen *via* MHC class II, antigen processing and presentation of peptide antigen, *etc.* (**Figure 7D**). In addition, the KEGG enrichment analysis found that upregulated differentially genes were enriched in Fc gamma R-mediated phagocytosis and antigen processing and presentation pathways (**Figure 7E**). Therefore, the differential genes of the two groups are involved in the pathway of antigen presentation, which also indicates that the expression of SIGLEC9 gene is related to the ability of macrophages to process antigens.

We also extracted macrophages (proliferating) for analysis and, as shown in **Supplementary Figure S2**, we found that SIGLEC9 was positively correlated with the proliferation genes (ANAPC11, CCNB1, and PLK1) (*P* < 0.05) of macrophages. According to the median of SIGLEC9 expression, we further divided the macrophages (proliferating) into two groups with a high expression and a low expression of SIGLEC9, respectively. We found the differential genes between the high-SIGLEC9-expression group and the low-SIGLEC9-expression group. The GSEA analysis found that upregulated genes were associated with macrophage maturation and M2 polarization (17). The GO enrichment found that upregulated genes were significantly enriched in the regulation of cell cycle phase transition, DNA replication, T cell activation, and other pathways, which are related to cell proliferation and replication. In addition, the KEGG enrichment found that upregulated genes were significantly enriched in pathways, such as DNA replication and cell cycle, which are also related to cell proliferation and replication. It indicates that the high expression of SIGLEC9 is related to the M2 macrophage polarization and proliferation of macrophages.

## Discussion

Glioma is a broad term of tumors occurring in the brain and spinal cord (18). There are more than 100 different pathological types of central nervous system and brain tumors, in which gliomas represent the largest proportion. The prognosis of gliomas after diagnosis varies significantly with the tumor grades, subtypes, and molecular biomarkers (19). The high-grade (III and IV) gliomas have a poor survival time. GBM also has poor overall survival, and the average length of survival after diagnosis is only 12 to 15 months. In the four molecular subtypes of GBM, the mesenchymal subtype tends to have the poorest overall survival than the other three subtypes (20). IDH is an enzyme that catalyzes the oxidative decarboxylation of isocitrate, and IDH wild-type glioma patients have a poor prognosis (21). The alpha thalassemia/intellectual disability syndrome X-linked (ATRX) gene is involved in telomere maintenance, and the loss of ATRX could reduce the median survival of glioma patients by promoting tumor growth (22).

In this study, we comprehensively analyzed the expression pattern of *SIGLEC9* in gliomas with TCGA and CGGA databases. High-*SIGLEC9*-expression patients had a shorter survival probability than low-*SIGLEC9*-expression patients. In addition, *SIGLEC9* expression was significantly upregulated in malignant pathological types such as grade III, grade IV, mesenchymal subtype, and IDH wild-type gliomas. Then, we investigated the protein levels of SIGLEC9 in 177 glioma patients from Sanbo Brain Hospital Capital Medical University. The results indicated that high-SIGLEC9-expression patients had a shorter survival probability than low-SIGLEC9-expression patients. In addition, high SIGLEC9 expression presented to have a shorter survival probability in patients with age ≧60 years, with grade IV glioma, with GBM, with ATRX loss glioma, without radiotherapy, or without chemotherapy. These parameters are all correlated with the poor overall survival of glioma patients as mentioned above. These results showed that SIGLEC9 expression was positively correlated with a malignant biologic process, indicating that SIGLEC9 might play important roles in the progression of gliomas.

Then, we investigated the underlying mechanisms of SIGLEC9 in gliomas, and our results showed that SIGLEC9 might regulate the tumor microenvironment (TME) in gliomas. TME is a dynamic condition in such a way that different immune cells interplay with cancer cells (23). TME has different inflammatory mediators, extracellular matrix, and signaling molecules to induce tumor progression and therapy resistance (24, 25). Tumor-infiltrated immune cells contain different types of cells, such as MDSCs, neutrophils, macrophages, dendritic cells, regulatory T cells (Treg), *etc.* (26, 27). Our results revealed that tumor-related immune cells, such as MDSCs, regulatory T cells, and neutrophils, were positively correlated with *SIGLEC9* expression in gliomas. *SIGLEC9* expression was mostly correlated with MDSC infiltration in glioma. MDSCs are a heterogenous group of immune cells with immature myeloid cells, including precursors of granulocytes, macrophages, and dendritic cells (28). MDSCs are strongly expanded in the site of cancer, such as gliomas, and have been demonstrated to be correlated with a poor prognosis and therapy resistance in cancer patients (29). MDSCs have strong abilities to exacerbate gliomas. Firstly, MDSCs induce the production of reactive oxygen species, nitric oxide, and arginase (30). Secondly, MDSCs could induce the maturity and development of tumor-induced regulatory T cells (31). Thirdly, MDSCs could increase the expression of prostaglandin E2 and cyclooxygenase 2 (32). These inflammatory mediators produced by MDSCs could increase the complexity of immune cell interactions in the TME of gliomas. Consistently, our data also showed that SIGLEC9 was positively correlated with the reactive oxygen species pathway in the GSEA analysis, and SIGLEC9 expression was positively correlated with regulatory T cell infiltration. Furthermore, SIGLEC9 expression was also positively correlated with immune checkpoints, including LAIR1, HAVCR2, CD86, and LGALS9. As an important member of the immune tumor microenvironment, macrophages play an important therapeutic role in glioma. Hara et al. have proven that macrophages can induce a transition of glioblastoma cells into mesenchymal-like (MES-like) states (33). They found that the state of MES-like glioblastoma is related to the increase in the expression of the medium germination program in the middle of macrophages, which has potential treatment for the widespread changes in the immune microenvironment. In the results of our single-cell sequencing analysis, compared with normal samples, we found that SIGLEC9 expresses highly in three types of macrophages [macrophage (proliferating), macrophage, and microglia/microphage] in tumor samples. Furthermore, we found that the SIGLEC9 gene is related to the ability of macrophages to process antigens and the proliferation of macrophages. In the future, we will demonstrate the regulatory role of SIGLEC9 in macrophages through further experiments. Thus, SIGLEC9 was considered to exacerbate the gliomas by suppressing the anti-tumor immune response.

Functional enrichment analysis showed that the *SIGLEC9*-correlated genes were enriched in neutrophil immune response. Neutrophils are the most abundant granulocytes to comprise approximately 70% of leukocytes in the peripheral blood of humans. Previous studies have revealed that the number of neutrophils is positively correlated with the severity and poor prognosis of glioma patients (34). In high-grade gliomas, neutrophilia was associated with poor survival to decreased overall survival (35). Cytokine granulocyte-colony stimulating factor (G-CSF) is the growth factor of neutrophils, and G-CSF is over-produced in glioma patients (36). In addition, G-CSF is responsible for the high neutrophil–lymphocyte ratio (NLR) in glioma patients *via* switching bone marrow hematopoiesis from lymphocytes to granulocytes (37). A high neutrophil/lymphocyte ratio is correlated with a poor prognosis of glioma patients. Neutrophils are also related to the resistance of anti-angiogenic therapy, such as anti-vascular endothelial growth factor therapy of glioma patients (38). Tumor-infiltrated neutrophils can secrete elastase to promote the proliferation of glioma cells (39).

In summary, SIGLEC9 might regulate the TME in gliomas to exacerbate the disease, and MDSCs and neutrophils play an important role in the function of SIGLEC9. Moreover, SIGLEC9 might upregulate the expression of immune checkpoint genes to suppress the anti-tumor immune response in gliomas.

## Conclusion

In this study, we analyzed the expression patterns and prognostic values of SIGLEC9 in glioma. *SIGLEC9* expression was significantly upregulated in malignant pathological types, such as grade III, grade IV, mesenchymal subtype, and IDH wild-type gliomas in TCGA and CGGA database. High *SIGLEC9* expression presented to have a shorter survival probability than low *SIGLEC9* expression in glioma patients. Our own clinical data also showed that high SIGLEC9 protein levels presented to have a shorter survival probability than low SIGLEC9 protein levels in patients with age ≧60 years, grade IV glioma, GBM, ATRX loss glioma, without radiotherapy, or without chemotherapy, which are all poor prognosis factors of gliomas. Furthermore, we investigated the underlying functions of SIGLEC9 in glioma pathogenesis, and we found that SIGLEC9 might regulate the TME to induce tumor growth, metastasis, and the therapy resistance of gliomas. We inferred that MDSCs and neutrophils might play an important role in the function of SIGLEC9. Moreover, SIGLEC9 might upregulate the expression of immune checkpoint genes to suppress the anti-tumor immune response in gliomas. These results indicated that high SIGLEC9 expression might serve as a poor prognosis marker for glioma patients and SIGLEC9 might be a therapeutic target for glioma in the future.

## Data Availability Statement

The original contributions presented in the study are included in the article/**Supplementary Material**. Further inquiries can be directed to the corresponding authors.

## Ethics Statement

The studies involving human participants were reviewed and approved by the ethics committee of Sanbo Brain Hospital Capital Medical University (no. SBNK-2018-003-01). Written informed consent to participate in this study was provided by the participants’ legal guardian/next of kin. Written informed consent was obtained from the individuals for the publication of any potentially identifiable images or data included in this article.

## Author Contributions

HX and YaF performed the statistical analysis and drafted the manuscript. WK, HW, and YuF contributed to database building. JZ, KY, and GH conceived the design of the study and revised the manuscript. All authors contributed to the article and approved the submitted version.

## Conflict of Interest

The authors declare that the research was conducted in the absence of any commercial or financial relationships that could be construed as a potential conflict of interest.

## Publisher’s Note

All claims expressed in this article are solely those of the authors and do not necessarily represent those of their affiliated organizations, or those of the publisher, the editors and the reviewers. Any product that may be evaluated in this article, or claim that may be made by its manufacturer, is not guaranteed or endorsed by the publisher.

## References

[1] Davis ME. Epidemiology and Overview of Gliomas. Semin Oncol Nurs (2018) 34(5):420–9. doi: 10.1016/j.soncn.2018.10.001 30392758

[2] Cahill D Turcan S. Origin of Gliomas. Semin Neurol (2018) 38(1):5–10. doi: 10.1055/s-0038-1635106 29548046

[3] Liu K Jiang Y. Polymorphisms in DNA Repair Gene and Susceptibility to Glioma: A Systematic Review and Meta-Analysis Based on 33 Studies With 15 SNPs in 9 Genes. Cell Mol Neurobiol (2017) 37(2):263–74. doi: 10.1007/s10571-016-0367-y PMC11482202 27055523

[4] Omuro A DeAngelis LM. Glioblastoma and Other Malignant Gliomas: A Clinical Review. JAMA (2013) 310(17):1842–50. doi: 10.1001/jama.2013.280319 24193082

[5] Wang Q Hu B Hu X Kim H Squatrito M Scarpace L. Tumor Evolution of Glioma-Intrinsic Gene Expression Subtypes Associates With Immunological Changes in the Microenvironment. Cancer Cell (2018) 33(1):152. doi: 10.1016/j.ccell.2017.12.012 29316430 PMC5892424

[6] Ghotme KA Barreto GE Echeverria V Gonzalez J Bustos RH Sanchez M. Gliomas: New Perspectives in Diagnosis, Treatment and Prognosis. Curr Top Med Chem (2017) 17(12):1438–47. doi: 10.2174/1568026617666170103162639 28049399

[7] Chen R Smith-Cohn M Cohen AL Colman H. Glioma Subclassifications and Their Clinical Significance. Neurotherapeutics (2017) 14(2):284–97. doi: 10.1007/s13311-017-0519-x PMC5398991 28281173

[8] Picca A Berzero G Di Stefano AL Sanson M. The Clinical Use of IDH1 and IDH2 Mutations in Gliomas. Expert Rev Mol Diagn (2018) 18(12):1041–51. doi: 10.1080/14737159.2018.1548935 30427756

[9] Kim ST Chu Y Misoi M Suarez-Almazor ME Tayar JH Lu H. Distinct Molecular and Immune Hallmarks of Inflammatory Arthritis Induced by Immune Checkpoint Inhibitors for Cancer Therapy. Nat Commun (2022) 13(1):1970. doi: 10.1038/s41467-022-29539-3 35413951 PMC9005525

[10] Bailey CM Liu Y Liu M Du X Devenport M Zheng P. Targeting HIF-1α Abrogates PD-L1-Mediated Immune Evasion in Tumor Microenvironment But Promotes Tolerance in Normal Tissues. J Clin Invest (2022) 132(9):e150846. doi: 10.1172/JCI150846 35239514 PMC9057613

[11] Fraschilla I Pillai S. Viewing Siglecs Through the Lens of Tumor Immunology. Immunol Rev (2017) 276(1):178–91. doi: 10.1111/imr.12526 PMC5860639 28258691

[12] Ibarlucea-Benitez I Weitzenfeld P Smith P Ravetch JV. Siglecs-7/9 Function as Inhibitory Immune Checkpoints *In Vivo* and Can Be Targeted to Enhance Therapeutic Antitumor Immunity. Proc Natl Acad Sci USA (2021) 118(26):e2107424118. doi: 10.1073/pnas.2107424118 34155121 PMC8256000

[13] Haas Q Boligan KF Jandus C Schneider C Simillion C Stanczak MA. Siglec-9 Regulates an Effector Memory CD8^+^ T-Cell Subset That Congregates in the Melanoma Tumor Microenvironment. Cancer Immunol Res (2019) 7(5):707–18. doi: 10.1158/2326-6066.CIR-18-0505 30988027

[14] Stanczak MA Siddiqui SS Trefny MP Thommen DS Boligan KF von Gunten S. Self-Associated Molecular Patterns Mediate Cancer Immune Evasion by Engaging Siglecs on T Cells. J Clin Invest (2018) 128(11):4912–23. doi: 10.1172/JCI120612 PMC6205408 30130255

[15] Beatson R Tajadura-Ortega V Achkova D Picco G Tsourouktsoglou TD Klausing S. The Mucin MUC1 Modulates the Tumor Immunological Microenvironment Through Engagement of the Lectin Siglec-9. Nat Immunol (2016) 17(11):1273–81. doi: 10.1038/ni.3552 PMC5257269 27595232

[16] De Freitas A Banerjee S Xie N Cui H Davis KI Friggeri A. Identification of TLT2 as an Engulfment Receptor for Apoptotic Cells. J Immunol (2012) 188(12):6381–8. doi: 10.4049/jimmunol.1200020 PMC3370132 22573805

[17] Pello OM De Pizzol M Mirolo M Soucek L Zammataro L Amabile A. Role of C-MYC in Alternative Activation of Human Macrophages and Tumor-Associated Macrophage Biology. Blood (2012) 119(2):411–21. doi: 10.1182/blood-2011-02-339911 22067385

[18] Ostrom QT Gittleman H Stetson L Virk SM Barnholtz-Sloan JS. Epidemiology of Gliomas. Cancer Treat Res (2015) 163:1–14. doi: 10.1007/978-3-319-12048-5_1 25468222

[19] Reifenberger G Wirsching HG Knobbe-Thomsen CB Weller M. Advances in the Molecular Genetics of Gliomas - Implications for Classification and Therapy. Nat Rev Clin Oncol (2017) 14(7):434–52. doi: 10.1038/nrclinonc.2016.204 28031556

[20] Behnan J Finocchiaro G Hanna G. The Landscape of the Mesenchymal Signature in Brain Tumours. Brain (2019) 142(4):847–66. doi: 10.1093/brain/awz044 PMC6485274 30946477

[21] Masui K Mischel PS Reifenberger G. Molecular Classification of Gliomas. Handb Clin Neurol (2016) 134:97–120. doi: 10.1016/B978-0-12-802997-8.00006-2 26948350

[22] Koschmann C Calinescu AA Nunez FJ Mackay A Fazal-Salom J Thomas D. ATRX Loss Promotes Tumor Growth and Impairs Nonhomologous End Joining DNA Repair in Glioma. Sci Transl Med (2016) 8(328):328ra28. doi: 10.1126/scitranslmed.aac8228 PMC5381643 26936505

[23] Wu T Dai Y. Tumor Microenvironment and Therapeutic Response. Cancer Lett (2017) 387:61–8. doi: 10.1016/j.canlet.2016.01.043 26845449

[24] Landskron G de la Fuente M Thuwajit P Thuwajit C Hermoso MA. Chronic Inflammation and Cytokines in the Tumor Microenvironment. J Immunol Res (2014) 2014:149185. doi: 10.1155/2014/149185 24901008 PMC4036716

[25] Gieryng A Pszczolkowska D Walentynowicz KA Rajan WD Kaminska B. Immune Microenvironment of Gliomas. Lab Invest (2017) 97(5):498–518. doi: 10.1038/labinvest.2017.19 28287634

[26] Elliott LA Doherty GA Sheahan K Ryan EJ. Human Tumor-Infiltrating Myeloid Cells: Phenotypic and Functional Diversity. Front Immunol (2017) 8:86. doi: 10.3389/fimmu.2017.00086 28220123 PMC5292650

[27] Boussiotis VA Charest A. Immunotherapies for Malignant Glioma. Oncogene (2018) 37(9):1121–41. doi: 10.1038/s41388-017-0024-z PMC5828703 29242608

[28] Kohanbash G Okada H. Myeloid-Derived Suppressor Cells (MDSCs) in Gliomas and Glioma-Development. Immunol Invest (2012) 41(6-7):658–79. doi: 10.3109/08820139.2012.689591 23017140

[29] Ding AS Routkevitch D Jackson C. Targeting Myeloid Cells in Combination Treatments for Glioma and Other Tumors. Front Immunol (2019) 10:1715. doi: 10.3389/fimmu.2019.01715 31396227 PMC6664066

[30] Youn JI Gabrilovich DI. The Biology of Myeloid-Derived Suppressor Cells: The Blessing and the Curse of Morphological and Functional Heterogeneity. Eur J Immunol (2010) 40(11):2969–75. doi: 10.1002/eji.201040895 PMC3277452 21061430

[31] Huang B Pan PY Li Q Sato AI Levy DE Bromberg J. Gr-1+CD115+ Immature Myeloid Suppressor Cells Mediate the Development of Tumor-Induced T Regulatory Cells and T-Cell Anergy in Tumor-Bearing Host. Cancer Res (2006) 66(2):1123–31. doi: 10.1158/0008-5472.CAN-05-1299 16424049

[32] Won WJ Deshane JS Leavenworth JW Oliva CR Griguer CE. Metabolic and Functional Reprogramming of Myeloid-Derived Suppressor Cells and Their Therapeutic Control in Glioblastoma. Cell Stress (2019) 3(2):47–65. doi: 10.15698/cst2019.02.176 31225500 PMC6551710

[33] Hara T Chanoch-Myers R Mathewson ND Myskiw C Atta L Bussema L.. Interactions Between Cancer Cells and Immune Cells Drive Transitions to Mesenchymal-Like States in Glioblastoma. Cancer Cell (2021) 39(6):779–792.e11. doi: 10.1016/j.ccell.2021.05.002 34087162 PMC8366750

[34] Gabrusiewicz K Rodriguez B Wei J Hashimoto Y Healy LM Maiti SN. Glioblastoma-Infiltrated Innate Immune Cells Resemble M0 Macrophage Phenotype. JCI Insight (2016) 1(2):85841. doi: 10.1172/jci.insight.85841 26973881 PMC4784261

[35] Schernberg A Nivet A Dhermain F Ammari S Escande A Pallud J. Neutrophilia as a Biomarker for Overall Survival in Newly Diagnosed High-Grade Glioma Patients Undergoing Chemoradiation. Clin Transl Radiat Oncol (2018) 10:47–52. doi: 10.1016/j.ctro.2018.04.002 29928705 PMC6008628

[36] Nitta T Sato K Allegretta M. Expression of Granulocyte Colony Stimulating Factor and G6ranulocyte-Macrophage Colony Stimulating Factor Genes in Human Astrocytoma Cell Lines and in Glioma Specimens. Brain Res (1992) 571(1):19–25. doi: 10.1016/0006-8993(92)90505-4 1377084

[37] Albulescu R Codrici E Popescu ID Mihai S Necula LG Petrescu D. Cytokine Patterns in Brain Tumour Progression. Mediators Inflamm (2013) 2013:979748. doi: 10.1155/2013/979748 23864770 PMC3707225

[38] Achyut BR Shankar A Iskander AS Ara R Angara K Zeng P. Bone Marrow Derived Myeloid Cells Orchestrate Antiangiogenic Resistance in Glioblastoma Through Coordinated Molecular Networks. Cancer Lett (2015) 369(2):416–26. doi: 10.1016/j.canlet.2015.09.004 PMC4686232 26404753

[39] Iwatsuki K Kumara E Yoshimine T Nakagawa H Sato M Hayakawa T. Elastase Expression by Infiltrating Neutrophils in Gliomas. Neurol Res (2000) 22(5):465–8. doi: 10.1080/01616412.2000.11740701 10935217

