# Supplementary material for: High Expression Levels of SIGLEC9 Indicate Poor Outcomes of Glioma and Correlate With Immune Cell Infiltration
Source: Front Oncol. 2022 Jun 9;12:878849. doi: 10.3389/fonc.2022.878849 (PMC9218569; doi:10.3389/fonc.2022.878849)
Supplement: Supplementary file 1 [file DataSheet_1.docx]

**1. Survival analysis of SIGLEC9 in different glioma subgroups.**

As can be seen from Figure S1, glioma patients were divided into SIGLEC9 high and low groups according to the median of SIGLEC9 expression, and the survival prognosis of SIGLEC9-high and SIGLEC9-low expression groups was significantly different in different glioma subgroups, such as Female (HR = 2,94, P < 0.001), Male (HR = 3.35, P < 0.001), Age ≤ 60 (HR = 2.96, P < 0.001), Age > 60 (HR = 2.46, P < 0.001), Mut (HR = 1.84, P = 0.007), WT (HR = 2.15, P < 0.001), Glioblastoma (HR = 1.48, P = 0.025), Oligodendroglioma (HR =1.99, P = 0.021), G2 (HR = 2.27, P = 0.028).

**
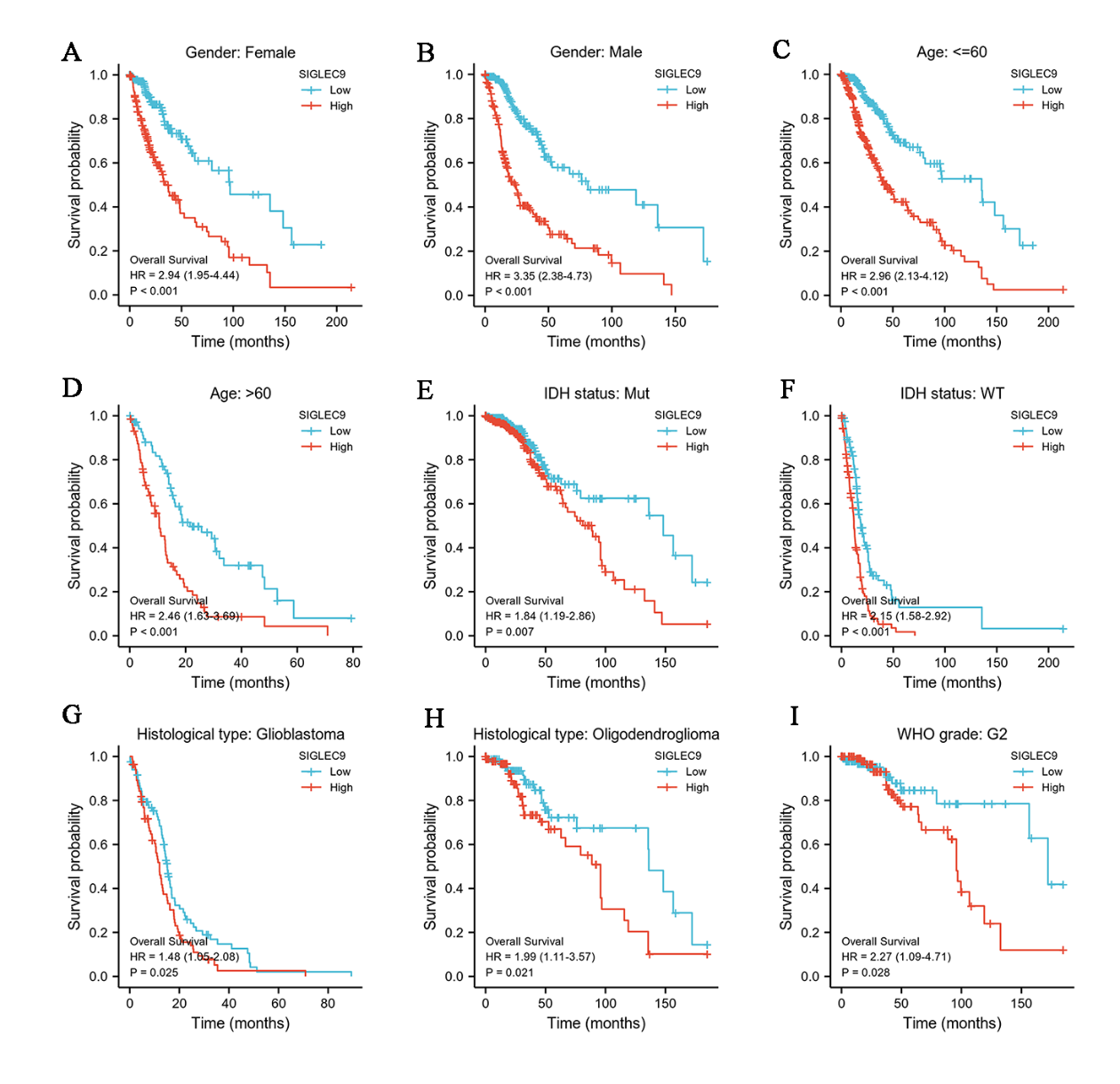
**

**Figure S1.** Survival analysis of SIGLEC9 in different glioma subgroups (Female, Male, Age ≤ 60, Ag > 60, Mut, WT, Glioblastoma, Oligodendroglioma, G2). **Abbreviation:** WT: Wild Type (IDH WT-codel); Mut: mutants (IDH Mut -codel); G2: grade 2.

**2. Regulatory Role of SIGLEC9 in Macrophages (Proliferating) With Glioma.**


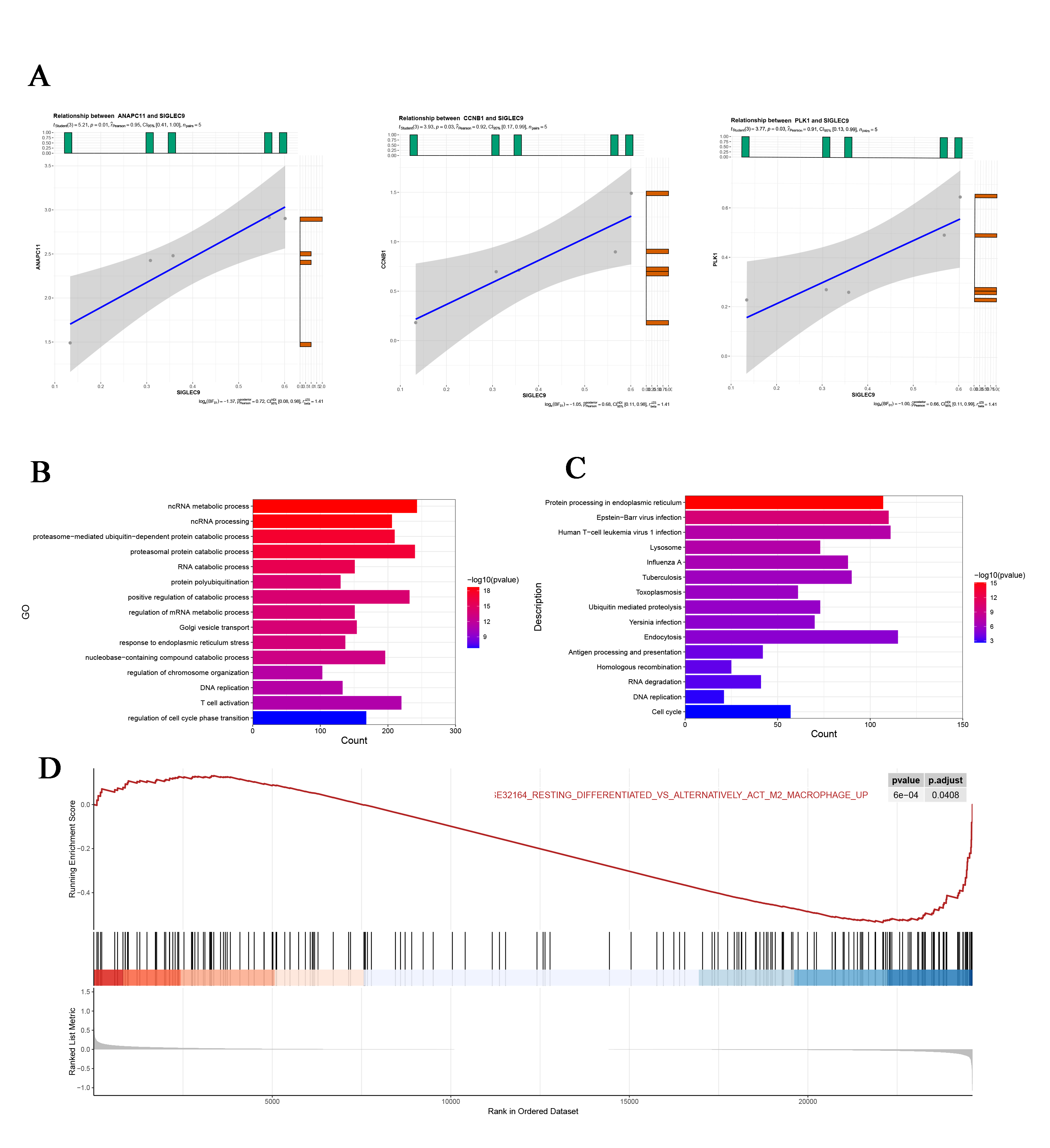


**Figure S2.** Regulatory role of SIGLEC9 in macrophages (proliferating) with glioma. (A) Correlation between SIGLEC9 and proliferating genes (ANAPC11, CCNB1, PLK1). (B) GO analysis between SIGLEC9 high and low groups in macrophages (proliferating). (C) KEGG analysis between SIGLEC9 high and low groups in macrophages (proliferating). (D) GSEA analysis between SIGLEC9 high and low groups in macrophages (proliferating).

**3. Baseline datasheet of 177 glioma patients.**

**Table S1:** Baseline datasheet of 177 glioma patients.

|  | **High- SIGLEC9 (N=26)** | **Low- SIGLEC9 (N=25)** | **medium- SIGLEC9 (N=126)** | **Overall (N=177)** | **P** |
| --- | --- | --- | --- | --- | --- |
| **Age** |  |  |  |  | **0.048** |
| <60 | 19 (73.1%) | 21 (84.0%) | 114 (90.5%) | 154 (87.0%) |  |
| ≥60 | 7 (26.9%) | 4 (16.0%) | 12 (9.5%) | 23 (13.0%) |  |
| **Gender** |  |  |  |  | 0.585 |
| **Female** | **12 (46.2%)** | 8 (32.0%) | 50 (39.7%) | 70 (39.5%) |  |
| **Male** | **14 (53.8%)** | 17 (68.0%) | 76 (60.3%) | 107 (60.5%) |  |
| **Pathology** |  |  |  |  | 0.057 |
| GBM | 24 (92.3%) | 19 (76.0%) | 88 (69.8%) | 131 (74.0%) |  |
| L | 2 (7.7%) | 6 (24.0%) | 38 (30.2%) | 46 (26.0%) |  |
| **IDH R132H** |  |  |  |  | 0.246 |
| MUT | 4 (15.4%) | 6 (24.0%) | 39 (31.0%) | 49 (27.7%) |  |
| WT | 22 (84.6%) | 19 (76.0%) | 87 (69.0%) | 128 (72.3%) |  |
| **Grade** |  |  |  |  | 0.161 |
| 2 | 2 (7.7%) | 1 (4.0%) | 13 (10.3%) | 16 (9.0%) |  |
| 3 | 0 (0%) | 5 (20.0%) | 25 (19.8%) | 30 (16.9%) |  |
| 4 | 24 (92.3%) | 19 (76.0%) | 88 (69.8%) | 131 (74.0%) |  |
| **event** |  |  |  |  | 0.358 |
| Live | 10 (38.5%) | 13 (52.0%) | 68 (54.0%) | 91 (51.4%) |  |
| Death | 16 (61.5%) | 12 (48.0%) | 58 (46.0%) | 86 (48.6%) |  |
| **ATRX** |  |  |  |  | 0.352 |
| high | 4 (15.4%) | 7 (28.0%) | 25 (19.8%) | 36 (20.3%) |  |
| low | 22 (84.6%) | 16 (64.0%) | 97 (77.0%) | 135 (76.3%) |  |
| none | 0 (0%) | 2 (8.0%) | 4 (3.2%) | 6 (3.4%) |  |
| **OS time (day)** |  |  |  |  | 0.738 |
| > 496 | 10 (38.5%) | 10 (40.0%) | 67 (53.2%) | 87 (49.2%) |  |
| ≤ 496 | 16 (61.5%) | 15 (60.0%) | 59 (46.8%) | 90 (50.8%) |  |
| **PDL1** |  |  |  |  | 0.293 |
| High | 14 (53.8%) | 10 (40.0%) | 47 (37.3%) | 71 (40.1%) |  |
| Low | 12 (46.2%) | 15 (60.0%) | 79 (62.7%) | 106 (59.9%) |  |
| **PDGFa** |  |  |  |  | 0.497 |
| low | 23 (88.5%) | 22 (88.0%) | 114 (90.5%) | 159 (89.8%) |  |
| none | 3 (11.5%) | 3 (12.0%) | 7 (5.6%) | 13 (7.3%) |  |
| high | 0 (0%) | 0 (0%) | 5 (4.0%) | 5 (2.8%) |  |

**Abbreviation:** PDGFa: Platelet Derived Growth Factor Subunit A; ATRX: ATRX Chromatin Remodeler; IDH: isocitrate dehydrogenase.

**4. Multivariate cox regression analysis of 177 glioma patients.**

As shown in Table S2, we performed multivariate cox regression analysis on 177 glioma patients. In a multivariate cox regression model, high-SIGLEC9 expression was an independent prognostic factor.

**Table S2. Multivariate cox regression analysis of 177 glioma patients**

| Characteristics | Total(N) | Multivariate analysis | |
| --- | --- | --- | --- |
|  |  | Hazard ratio (95% CI) | P value |
| **Age** | 177 |  |  |
| <60 | 154 |  |  |
| ≥60 | 23 | 0.295 (0.090-0.968) | **0.044** |
| **Gender** | 177 |  |  |
| Male | 107 |  |  |
| Female | 70 | 3.353 (1.194-9.417) | **0.022** |
| **Pathology** | 177 |  |  |
| L | 46 |  |  |
| GBM | 131 | 1.365 (0.301-6.192) | 0.686 |
| **IDH_R132H** | 177 |  |  |
| WT | 128 |  |  |
| MUT | 49 | 4.737 (2.039-11.006) | **< 0.001** |
| **Grade** | 177 | 0.754 (0.160-3.558) | 0.754 |
| **ATRX** | 177 |  |  |
| low | 135 |  |  |
| high | 36 | 0.845 (0.432-1.652) | 0.622 |
| none | 6 | 0.146 (0.017-1.267) | 0.081 |
| **PDL1** | 177 |  |  |
| Low | 106 |  |  |
| High | 71 | 2.071 (1.235-3.473) | **0.006** |
| **PDGFa** | 177 |  |  |
| low | 159 |  |  |
| none | 13 | 0.107 (0.011-1.017) | 0.052 |
| high | 5 | 0.174 (0.062-0.487) | **0.001** |
| **Total.Area** | 177 | 1(1.000-1.000) | 0.730 |
| **SIGLEC9** | 177 |  |  |
| Low | 25 |  |  |
| High | 26 | 4.019 (1.113 - 14.512) | **0.034** |
| Medium | 126 | 0.835 (0.422 – 1.652) | 0.604 |

**5. Differences in immune cell infiltration between high and low expression of SIGLEC9**

**Table S3.** Differences in immune cell infiltration between high and low expression of SIGLEC9

| Group | Group [counts] | Group [counts] | Statisticst | degree of freedom (df) | Difference (J-I) | confidence interval (95%CI) | p-value |
| --- | --- | --- | --- | --- | --- | --- | --- |
| T cells | High [349] | Low [349] | -11.310 | 617.4982 | -0.058 | -0.068 - -0.048 | 0.000 |
| NK cells | High [349] | Low [349] | -6.118 | 675.8160 | -0.008 | -0.011 - -0.005 | 0.000 |
| pDC | High [349] | Low [349] | 7.161 | 695.9992 | 0.051 | 0.037 - 0.065 | 0.000 |
| Neutrophils | High [349] | Low [349] | -21.193 | 605.3497 | -0.084 | -0.091 - -0.076 | 0.000 |
| aDC | High [349] | Low [349] | -17.230 | 667.8419 | -0.101 | -0.112 - -0.089 | 0.000 |
| B cells | High [349] | Low [349] | -4.501 | 663.7717 | -0.013 | -0.019 - -0.007 | 0.000 |
| CD8 T cells | High [349] | Low [349] | -0.495 | 690.4077 | 0.000 | -0.002 - 0.001 | 0.621 |
| DC | High [349] | Low [349] | -3.969 | 655.9750 | -0.022 | -0.033 - -0.011 | 0.000 |
| iDC | High [349] | Low [349] | -15.872 | 658.0245 | -0.043 | -0.048 - -0.037 | 0.000 |
| Macrophages | High [349] | Low [349] | -24.419 | 622.2769 | -0.085 | -0.092 - -0.078 | 0.000 |
| Mast cells | High [349] | Low [349] | -2.167 | 693.1183 | -0.007 | -0.014 - -0.001 | 0.031 |
| T helper cells | High [349] | Low [349] | -4.892 | 694.1106 | -0.006 | -0.008 - -0.004 | 0.000 |
| Th1 cells | High [349] | Low [349] | -7.215 | 679.0509 | -0.014 | -0.018 - -0.01 | 0.000 |
| Th2 cells | High [349] | Low [349] | -6.880 | 690.4086 | -0.021 | -0.026 - -0.015 | 0.000 |
| Th17 cells | High [349] | Low [349] | -7.431 | 678.7091 | -0.030 | -0.038 - -0.022 | 0.000 |
| TReg | High [349] | Low [349] | 3.974 | 664.2669 | 0.017 | 0.009 - 0.026 |  |
